# HYGIEIA: HYpothesizing the Genesis of Infectious Diseases and Epidemics through an Integrated Systems Biology Approach

**DOI:** 10.3390/v14071373

**Published:** 2022-06-23

**Authors:** Bradley Ward, Jean Cyr Yombi, Jean-Luc Balligand, Patrice D. Cani, Jean-François Collet, Julien de Greef, Joseph P. Dewulf, Laurent Gatto, Vincent Haufroid, Sébastien Jodogne, Benoît Kabamba, Sébastien Pyr dit Ruys, Didier Vertommen, Laure Elens, Leïla Belkhir

**Affiliations:** 1 Integrated Pharmacometrics, Pharmacogenomics and Pharmacokinetics Group (PMGK), Louvain Drug Research Institute (LDRI), UCLouvain, Université Catholique de Louvain, 1200 Brussels, Belgium; bradley.ward@uclouvain.be (B.W.); sebastien.pyrditruys@uclouvain.be (S.P.d.R.); 2 Louvain Center for Toxicology and Applied Pharmacology (LTAP), Institut de Recherche Expérimentale et Clinique (IREC), UCLouvain, Université Catholique de Louvain, 1200 Brussels, Belgium; julien.degreef@saintluc.uclouvain.be (J.d.G.); joseph.dewulf@saintluc.uclouvain.be (J.P.D.); vincent.haufroid@saintluc.uclouvain.be (V.H.); 3 Department of Internal Medicine, Cliniques Universitaires Saint-Luc, UCLouvain, Université Catholique de Louvain, 1200 Brussels, Belgium; jean.yombi@uclouvain.be; 4 WELBIO (Walloon Excellence in Life Sciences and Biotechnology), Pole of Pharmacology and Therapeutics (FATH), Institut de Recherche Experimentale et Clinique (IREC), Cliniques Universitaires Saint-Luc, UCLouvain, Université Catholique de Louvain, 1200 Brussels, Belgium; jean-luc.balligand@uclouvain.be; 5 WELBIO (Walloon Excellence in Life Sciences and Biotechnology), Metabolism and Nutrition Research Group, Louvain Drug Research Institute (LDRI), UCLouvain, Université Catholique de Louvain, 1200 Brussels, Belgium; patrice.cani@uclouvain.be; 6 WELBIO (Walloon Excellence in Life Sciences and Biotechnology), de Duve Institute, UCLouvain, Université Catholique de Louvain, 1200 Brussels, Belgium; jean-francois.collet@uclouvain.be; 7 Department of Laboratory Medicine, Cliniques Universitaires Saint-Luc, UCLouvain, Université Catholique de Louvain, 1200 Brussels, Belgium; benoit.kabamba@saintluc.uclouvain.be; 8 Department of Biochemistry, de Duve Institute, UCLouvain, Université Catholique de Louvain, 1200 Brussels, Belgium; 9 Computational Biology and Bioinformatics Unit (CBIO), de Duve Institute, UCLouvain, Université Catholique de Louvain, 1200 Brussels, Belgium; laurent.gatto@uclouvain.be; 10 Computer Science and Engineering Department (INGI), Institute of Information and Communication Technologies, Electronics and Applied Mathematics (ICTEAM), UCLouvain, Université Catholique de Louvain, 1348 Louvain-la-Neuve, Belgium; sebastien.jodogne@uclouvain.be; 11 Pôle de Microbiologie, Institut de Recherche Expérimentale et Clinique, UCLouvain, Université Catholique de Louvain, 1200 Brussels, Belgium; 12 De Duve Institute, and MASSPROT Platform, UCLouvain, Université Catholique de Louvain, 1200 Brussels, Belgium; didier.vertommen@uclouvain.be

**Keywords:** COVID-19, post COVID condition, proteomics, metabolomics, genomics, metagenomics, transcriptomics, network medicine

## Abstract

More than two years on, the COVID-19 pandemic continues to wreak havoc around the world and has battle-tested the pandemic-situation responses of all major global governments. Two key areas of investigation that are still unclear are: the molecular mechanisms that lead to heterogenic patient outcomes, and the causes of Post COVID condition (AKA Long-COVID). In this paper, we introduce the HYGIEIA project, designed to respond to the enormous challenges of the COVID-19 pandemic through a multi-omic approach supported by network medicine. It is hoped that in addition to investigating COVID-19, the logistics deployed within this project will be applicable to other infectious agents, pandemic-type situations, and also other complex, non-infectious diseases. Here, we first look at previous research into COVID-19 in the context of the proteome, metabolome, transcriptome, microbiome, host genome, and viral genome. We then discuss a proposed methodology for a large-scale multi-omic longitudinal study to investigate the aforementioned biological strata through high-throughput sequencing (HTS) and mass-spectrometry (MS) technologies. Lastly, we discuss how a network medicine approach can be used to analyze the data and make meaningful discoveries, with the final aim being the translation of these discoveries into the clinics to improve patient care.

## 1. Introduction

### 1.1. Clinical Problem and Phenotype Definition

COVID-19 is a multisystemic disease that is characterized by a complex heterogenous clinical pattern—the so called “infectious phenotype”. In the time since its initial outbreak in Wuhan at the end of 2019, the pathogen responsible for this disease, SARS-CoV-2, has spread to every country on Earth, infecting over 500 million people, and leading to the death of over 6 million people [1]. COVID-19 symptoms can range from asymptomatic/mild in about 80% of cases, to severe illness in around 5% of cases, potentially resulting in death (exact percentages depending on infectious variant and underlying immunity) [2]. Although vaccinations and improved patient care have greatly reduced the burden of this disease, fatality rates still remain extremely high with a global average of 138 deaths per 100,000 population; for reference, influenza, which is often compared to COVID-19, accounts for between 4.1 and 9.3 deaths per 100,000 [3,4].

#### 1.1.1. Susceptibility to SARS-CoV-2 and Clinical Presentations

Once SARS-CoV-2 infection is established, viral replication begins within the nasopharyngeal mucosa before spreading towards the lower respiratory tract. The trimeric spike (S) protein, which covers the surface of SARS-CoV-2, binds to the host cell receptor angiotensin-converting enzyme 2 (ACE-2) and mediates viral cell entry [5]. Through this mechanism, SARS-CoV-2 is capable of infecting host tissues within the lungs, where ACE-2 is highly expressed, as well as other tissues which express ACE-2 such as the heart, kidneys, liver, and brain [6,7].

Among symptomatic patients, the lungs are the organs most affected by the disease, causing respiratory failure which may progress to acute respiratory distress syndrome (ARDS) requiring mechanical ventilation. This is frequently accompanied by an overwhelming inflammatory reaction (cytokine storm) [8,9]. Risk factors of clinical severity and fatality have been identified and include older age and/or comorbidities, of which diabetes, obesity, hypertension, cardiovascular, and chronic kidney diseases are the most frequent [10]. Intriguingly, phenotype variability goes beyond severity: clinical data have promptly demonstrated that COVID-19 is a multisystem disease and can present with thrombo-embolic, kidney or neurological symptoms [11,12,13].

#### 1.1.2. Post COVID Condition

Most COVID-19 patients with mild to moderate disease progressions will usually recover after two to three weeks, but those patients presenting with severe disease usually take at least six weeks to recover. Around 54% and 34% of hospitalized and non-hospitalized patients respectively, will then continue to persist with COVID-19-related symptoms, most commonly including fatigue and muscle weakness, dyspnea, joint and chest pain, and neurocognitive impairment [14,15]. Interestingly, a literature report from the Belgian Health Care Knowledge Center showed an increasing trend of the incidence of displaying post COVID symptoms depending on study follow-up, with a median incidence of 17% (non-hospitalized patients) and 50.9% (hospitalized patients) at 1–3 months, increasing to 25% (non-hospitalized patients) and 62% (hospitalized patients) at 6 months+ follow up [16].

Several studies put the incidence of displaying persistent post COVID symptoms between 30% and 90% at 6 months past initial disease onset [17]. The pathogenesis of post COVID condition is still unclear, but it has been observed to more likely affect patients that suffered severe COVID-19 or those that required hospital admission [18]. A higher incidence is also seen in patients that presented with more than five symptoms during the acute phase of the disease, female patients, obese patients, and those patients with diabetes [19].

Recently, a multi-omic study with a focus on single-cell-omics by Su et al. [20] investigated post COVID conditions at between 2- and 3-months post COVID-19 diagnosis. They reported 61% of patients to have at least one symptom and also reported four main risk factors to develop post COVID symptoms: type 2 diabetes, reactivated EBV, auto-antibodies, and SARS-CoV-2 blood viral load. These risk factors have the potential to be used to predict patient risk to developing post COVID condition if measured at diagnosis. For example, around half of the patients exhibited auto antibodies at follow-up, also had them at diagnosis, yet the vast majority of these patients did not have any documented autoimmune conditions, suggesting the presence of a pre-existing subclinical condition in these patients. In addition to these risk factors, the study was also able to group patients into four distinct immune endpoints: type 1, type 2, intermediate, and naïve. These endpoints were characterized by unique immune system responses, COVID-19 disease severity, and risk of developing post COVID condition. However, one major drawback to this study was the lack of a genomic aspect, and so any links between host genomics and presence of auto-antibodies or patient immune endpoint groupings cannot be inferred.

In another study looking to identify predictors of post COVID condition, by Cervia et al. [21], a specific immunoglobulin signature during COVID-19 infection was identified in patients who later went on to develop post COVID condition. Using this signature in combination with certain clinical factors, the team were thus able to develop a post COVID prediction model for hospitalized patients.

These studies add to mounting research suggesting post COVID condition is a result of a mix of viral and host factors, such as the host microbiome or residual inflammation [22,23], and highlight the necessity of large scale multi-omic investigations that are able to consider host and viral factors in the context of the genomics, transcriptomics, proteomics, metabolomics, and metagenomics.

### 1.2. Previous Research and Gaps in Knowledge

The high morbidity and mortality rates of SARS-CoV-2 since the beginning of the pandemic have gradually fallen through a combination of vaccines, milder viral strains, and the rapid translation of research to clinical settings. Currently, most governments are relying on their vaccination programs to reduce the strain on their healthcare systems. Yet, with the continued emergence of new variants of concern presenting with antibody-escaping features, hospitalizations and reinfection risks continue to increase whilst vaccine efficacy decreases over time (especially against symptomatic infections), prompting governments to recommend “booster shots” in an effort to reinforce their populations immunity [24,25]. In consequence, there is an urgent need to identify and thoroughly map disease pathways at all levels: from the genome and metagenome, to the transcriptome, proteome, and metabolome, in order to elucidate SARS-CoV-2 specific therapeutic targets and biomarkers. Despite the global research effort into COVID-19 pathogenesis, most research tends to focus on single-omic datasets, such as the genome or proteome; even the few articles that have adopted a multi-omic design tend to only investigate two [26,27,28] or three [29,30,31] omic-levels. This has led to many gaps in our knowledge of COVID-19 pathogenic pathways, whereby parts of a pathway have been identified, but upstream and downstream consequences are still unknown. This project thus aims to fill these gaps using an unbiased approach to discover novel therapeutic targets and vaccination strategies as well as predictive/prognostic biomarkers.

#### 1.2.1. Viral Genome

Owing to its proofreading gene, *nsp14*, SARS-CoV-2 is characterized by a stable genome [32]. Nevertheless, as SARS-CoV-2 continues to circulate around the world, mutations and variants have emerged. Regarding some notable current and previous variants of concern (as of 01/06/22): Alpha (B.1.1.7), Beta (B.1.351), Gamma (P.1), and Delta (B.1.617.2) have all shown increased transmissibility and severity [33,34,35,36,37], whilst for the Omicron variants (BA.1 and BA.2), transmissibility is also increased, but severity appears to be reduced, especially among populations with a high level of immunity [38,39,40,41,42]. The most recently added variants, two more Omicron variants, BA.4 and BA.5, first detected in South Africa in January and February 2022 respectively, have since become the dominant variants there, while the Portuguese National Institute of Health estimated that BA.5 accounted for around 37% of positive cases in Portugal as of 8 May 2022, and the ECDC suggests it will likely become the dominant variant in Europe within the next few months [43]. The observed growth advantage of these lineages is likely due to improved immune evasion compared to previous omicron variants, resulting in increased transmissibility, but no observed impact on severity [44,45]. This appears to be in line with the virulence-transmissibility evolutionary trade-off theory whereby it is expected that the emergence of new dominating viral variants will favor increased transmissibility over virulence.

Initial research into viral variant pathogenicity seems to suggest they induce distinct humoral responses and transcriptional profiles [46,47]. However, more research is required in order to identify variant-specific molecules for therapeutic and sanitary countermeasures.

#### 1.2.2. Nasopharyngeal Microbiome

For COVID-19 patients, pathogenic respiratory co-infections have been found frequently in a number of studies, with between 7–14% of hospitalized patients presenting with bacterial co-infections [48,49]. For those patients admitted to the ICU, this number has been seen to rise up to 41% of patients presenting with secondary co-infections, although other studies have also noted lower numbers of 13.9% for ICU patients [50,51]. However, most studies only include targeted diagnosis and the majority tends to focus on bacteria, excluding co-infections of viruses, archaea, and fungal species which make up a sizeable proportion of the microbiome diversity.

Conversely, metagenomic high throughput sequencing (mHTS) has the ability to unbiasedly detect all microorganisms in a sample, providing extra information on the composition of the microbiome. Using such methods, the diversity of the respiratory microbiome in COVID-19 patients has been observed to decrease by 38% compared to healthy individuals, with a decrease in commensal bacteria and an increase of opportunistic pathogens [52]. Due to a lack of multi-omic studies investigating both host -omics and the microbiome, the consequences of this dysbiosis on COVID-19 progression and severity is yet to be fully characterized. It could be that opportunistic pathogens further exacerbate lung damage, or, it may be due to a reduction in commensal species involved in priming the innate immune system, weakening the patient’s immune response [53].

Further, recent evidence has found the microbiome to strongly influence the metabolome around the body and has been associated with cardiovascular disease, drug response, and asthma [54,55,56]. Specifically, to COVID-19, it has been seen that respiratory microbiome changes due to SARS-CoV-2 are associated with transcriptomic differences in several metabolic pathways [57]. This perhaps reflects metabolomic differences associated with COVID-19 pathogenesis and severity, but as no multi-omic studies have been conducted to investigate this link between the metabolome and microbiome, this currently remains speculation.

#### 1.2.3. Host Transcriptome

In COVID-19 patients, suppression of interferon (IFN) response has emerged as a major clinical determinant, with a complete loss of response associated with the most severe cases; a key differentiator from severe acute respiratory syndrome (SARS) and Middle East respiratory syndrome (MERS) [58,59,60]. The non-structural proteins NSP 1, 8, 9, and 16, have been indicated as partially responsible for this phenomenon via global suppression of mRNA splicing and translation, and via interfering with membrane trafficking of proteins [61]. Further, enhanced nuclear factor kappa-B (NF-ĸB) signaling, through a number of SARS-CoV-2 upregulated pathways, has been shown to lead to excessive inflammation, with inhibition of such molecular processes resulting in alleviation of severe symptoms [62,63,64]. However, more research is required to better understand these aforementioned pathways in the context of COVID-19 pathogenesis, particularly AGE-RAGE signaling which is associated with COVID-19 comorbidities and consequences like diabetes, inflammatory disease, and acute respiratory distress syndrome (ARDS) [63,65,66,67]. Also, of interest is the olfactory transduction pathway which is significantly activated during COVID-19 disease; dysfunction of this pathway (resulting in the loss of smell symptom) has been associated with faster recovery, perhaps due to the ultra-rapid antiviral response observed in olfactory receptor neurons [63,68,69,70]. This link begs further study, and may partially explain why older patients who tend to poses fewer such neurons, have a suppressed earlier antiviral response and higher disease severity in general.

Additional explanations for the increased severity seen in older populations come in the form of age-induced differences in gene expression. Both TMPRSS2 and ACE2, receptors recognized by the SARS-CoV-2 spike protein, show an increased expression with age in mammals, which may be partially responsible for the increased disease severity seen in these patients [71]. Further to this, a study by Mercatelli et al. [72], identified significant overlaps between SARS-CoV-2 interacting proteins and host age-related proteins, with viral infection affecting aging molecular mechanisms centered around eight proteins. Of these proteins, EEF2, NPM1, HMGA1, APEX1, and CHEK1, were found to have an age-dependent modulation in the lung tissues of males, whilst APEX1 was shown to have an age-dependent modulation in females. Such findings suggest a potential mechanism by which age-/sex-dependent severity of COVID-19 may manifest.

Other dysregulated genes include *ACO3*, *ATL3*, *S100A8*, *S100A9*, *IL-1B*, *IL-6*, *IL-8*, *TXNIP*, *ARRDC3*, *ACE-2*, and the *MHC-II* family of genes, and remain of interest due to their roles in cytokine storm development, inflammation, antigen presentation, viral replication, and immune evasion; 416 genes in total were seen to be deregulated specifically due to SARS-CoV-2 in a transcriptomic analysis [73,74,75,76,77,78]. This gene dysregulation is likely linked to adherent ncRNA dysregulation also seen in SARS-CoV-2 infections [79,80,81]. Viral transcript interaction with human ncRNA, as well as viral ncRNA, can enhance viral evasion of the immune system, enhance replication, promote transcript stability, and/or produce alternative transcripts to enhance virulence [82,83]. Specifically, *ORF-6*, *-7a*, and *-7b* contain complementary sequences to a number of ncRNAs involved in the innate/acquired immune response, antibody production, vaccine response, disease severity, and metabolic pathway activation [84,85,86,87,88]. The association of these viral transcripts to human ncRNAs has mainly been demonstrated in silico and remain to be studied in vivo/in vitro.

For further analysis of mRNA and ncRNA, multi-omic studies featuring transcriptomic and proteomic natures will be essential due to the lack of correlation between the two-omic levels, as observed in numerous studies [89,90,91,92,93,94,95].

#### 1.2.4. Host Proteomics and Metabolomics

The dysregulation seen within the genome, transcriptome, and microbiome, is also reflected in the proteome and metabolome. This dysregulation can lead to the identification of circulating biomarkers which play critical roles in clinical decision making. Indeed, a number of studies have investigated protein/metabolite biomarkers in relation to COVID-19, identified those that are differentially expressed, and presented molecular signatures that can either differentiate severity or predict progression [96]. However, although these molecular signatures tend to relate to similar pathways, such as platelet degranulation, complement, or coagulation, the specific protein/metabolite patterns in each study are almost never in agreement. This is perhaps related to the tendency of these studies to use lower patient numbers (ranging between 8 and 69) or to use targeted identification techniques [97,98,99,100,101,102,103,104,105]. Unbiased, shotgun mass spectrometry techniques with larger patient numbers could potentially overcome this discordance, and result in much more applicable biomarker patterns. Additionally, a multi-omic exploration of such a cohort would be able to identify biomarkers across all strata, further strengthening the credibility of such observed molecular patterns.

#### 1.2.5. Host Genomics

Numerous genome studies have been launched by a variety of institutions. From these studies, several genes/loci have been identified to impact the etiology of COVID-19; of note is the 3p21.31 locus, *OAS 1/2/3* located at 12q24.13, and the ABO loci which were highlighted early on in the COVID-19 pandemic [106]. Both 3p21.31 and 12q24.13 carry haplotypes of Neanderthals origin, which tend to be more difficult in terms of disentangling the causal genetic variants due to their size (often spanning tens of thousands of bases) [107,108].

The protective *OAS 1/2/3* alleles on 12q24.13, which confer around a 23% reduction in the risk of becoming critically ill from COVID-19 [109]. These genes encode enzymes catalyzing short polyadenylate synthesis, this subsequently activates ribonuclease L which degrades intracellular double-stranded RNA and triggers other antiviral mechanisms [110]. A medallion randomization study found higher levels of circulating OAS 1 levels were associated with the observed reduced risk, whilst other transcriptome-wide evidence suggested a stronger association with OAS 3 levels [111,112]. However, a recent study pinpoints to a SNP at rs10774671, located in a splice acceptor site at exon 7 of *OAS 1* [109]. Polymorphisms at this site determines the length of the protein encoded by *OAS 1*, with the protective allele (G) resulting in a longer and more active enzyme, increasing ribonuclease L activation and antiviral countermeasures.

Moving onto the locus 3p21.31, a major common risk factor is rs10490770. Carriers of the risk allele (C) are at increased risk of all-cause mortality and development of COVID-19 complications such as severe respiratory failure, venous thromboembolism, and hepatic injury [113]. An age dependent impact was also observed, with a more pronounced effect of the risk allele observed in individuals under 60 years of age. Importantly, this risk allele is commonly seen among European (allele frequency = 14.4%) and South Asian (allele frequency = 47.1%) populations, as well as in Admixed Americans, African, and East Asian populations to a lesser extent (allele frequency = 9.5%, 2.4%, and 0.4% respectively). Although specific causal links for 3p21.31 are not yet established, evidence points to variants of *SLC6A20* (which interacts with ACE2) and *CXCR6* (involved in T-cell recruitment) to explain the increased susceptibility and severity [114].

For the ABO locus, blood group O has been correlated with reduced susceptibility, perhaps attributed to anti-A IgG protection [106,115,116]. Other highlighted genetic risk factors include *INFAR2*, *DPP9*, *TYK2*, *ACE2*, and the *HLA* gene family [111,117,118]. These genes are all involved in immune signaling, antigen presentation, and/or cell entry receptors [119,120,121].

One of the largest genome sequencing studies, which investigated over 7000 critical COVID-19 cases and almost 50,000 controls was able to identify 16 new COVID-19 associated variants [122]. Five of these variants have direct roles in interferon signaling, this includes a probable destabilizing amino acid substitution in *IFNA10,* as well as another variant resulting in a reduction of a subunit of its receptor, *IFNAR2*. The results of the study provide robust evidence that reduced interferon signaling increases patient susceptibility to developing critical COVID-19. In addition to this, the study also identified variants of genes controlling levels of coagulation factor VIII to be associated with critical illness, which may explain some of the clotting abnormalities seen in severe COVID-19.

For most variants and genes associated with COVID susceptibility/severity, questions remain surrounding their causal links with the disease, and how changes within the genome reflect disease pathogenesis within the patient.

#### 1.2.6. Network Integration

Classical reductionism continues to be challenged amid mounting evidence of the importance of looking instead at the numerous interactions between biological components. Previously, such a narrow approach was demanded due to the limitations in data collection and analysis. Now however, multi-omic data can be integrated via computational networks and analyzed to better explain classification, improve predictions, or understand complex molecular pathways that would remain hidden for single-omic studies [123,124,125]. Song et al. [126] has demonstrated the benefits of such an approach in a study that identified two FDA-approved drugs suitable for repurposing to treat cardiovascular calcification, a pathology that has been under investigation for over 80 years. Previous attempts of this approach in the context of COVID-19 has resulted in improved therapeutic options, novel biomarkers, and enhanced pathophysiological knowledge, yet most studies featured low patient numbers and only included limited biological strata [28,29,30,104]. Below we present the largest, most detailed multi-omic analysis of a COVID-19 patient cohort to date, featuring data from all levels (host and viral genome, transcriptome, metagenome, proteome, and metabolome). Such an effort holds the promise of revealing novel components, crucial interactions, and emergent properties of this disease which would otherwise remain hidden. 

## 2. Materials and Methods

### 2.1. Cohort Population, Inclusion Criteria, and Sampling Methodology

**Figure 1:**
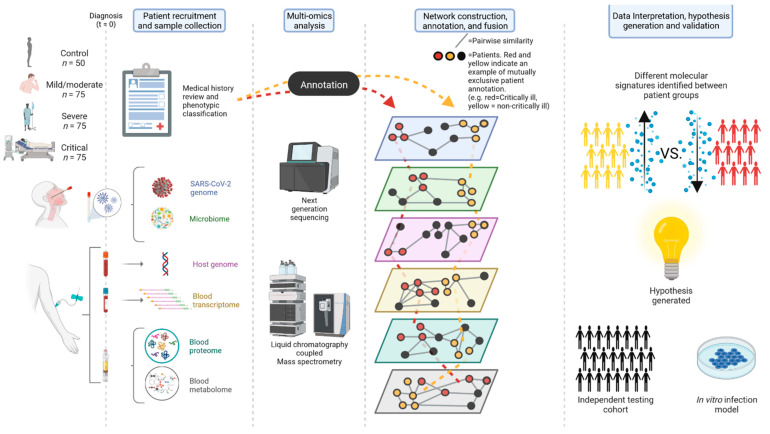
General scheme of the study, from patient recruitment at diagnosis, to -omics analysis, network fusion, data interpretation, and finally hypothesis generation and validation (through cohorts or infection models). (Created with BioRender.com©).

The general scheme of the clinical study and sample collection is depicted in Figure 1. Patient recruitment is currently ongoing with a planned 225 total patients and 50 total controls expected to be recruited from Cliniques Universitaires Saint-Luc (CUSL), Brussels, Belgium. In addition, patients are also being recruited from other hospitals in the Brussels and Wallonie region of Belgium in a multi-centric effort, and so total cohort size could potentially exceed this. The patient population will be split into three groups comprised of 75 individuals each: mild/moderate, severe, and critical. The control population will be split into two groups: 25 respiratory failure patients and 25 healthy individuals.

Patients will be >18 years old and provide informed consent. COVID-19 status will be determined via a SARS-CoV-2 RT-PCR test performed on nasopharyngeal (NP) swabs. Patient grouping will be based on CDC disease severity guidelines [127]. Respiratory failure controls should have a diagnosis of hypoxemic respiratory failure from an infectious origin (excluding SARS-CoV-2), and should not have tested SARS-CoV-2 positive within 6 months. Healthy controls should present without respiratory failure (i.e., SpO2 > 93%), and should not have tested SARS-CoV-2 positive within 6 months.

Biological samples will consist of whole EDTA blood, Tempus™ Blood RNA Tube, heparinized plasma, and NP swabs, taken in the first instance at the time of patient inclusion during the acute phase, and at the second instance around three months later. Later time points may be added for patients beyond three months, however, the principle focus of this project is the investigation of patients displaying post COVID condition at three months after diagnosis. All samples will be stored at −80 °C until patient recruitment is finished and the multi-omic analysis begins.

#### Sample Size Considerations

In this project, we guarantee complete -omics exploration of 225 patients with ideally, 75 each critically, severely and mildly/moderately ill patients and 50 controls. These realistic figures are based on sample inventory done while preparing this project and the ongoing patient recruitment rate at CUSL.

To compare this cohort size to similar multi-omic approaches used in the context of e.g., Alzheimer [128], cancer [129], or cardiovascular disease [130,131,132], we can see these studies tend to be characterized by smaller (*n* = 25, *n* = 63, *n* = 157) or slightly larger patient cohort sizes (*n* = 276, *n* = 364). In addition to this, COVID-19 multi-omic studies also tend to be characterized by both smaller cohort sizes (*n* = 14, *n* = 20, *n* = 102, *n* = 209), as well as the exploration of a reduced number of -omic strata in comparison to this proposed study [20,28,29,30]. Further, focusing specifically on post COVID condition, there is again a tendency for studies to either investigate smaller cohort sizes or cohorts of similar size (*n* = 103, *n* = 106, *n* = 121, *n* = 134, *n* = 143, *n* = 165, *n* = 215 [18,21,22,23,133,134,135]). Taken together, it is likely that the size and detail of this study will achieve not just similar, but also more significant results in comparison to these aforementioned studies.

### 2.2. Multi-Omic Analysis

All analyses will be conducted on patient samples collected at the point of diagnosis, as well as on patient samples collected during follow-ups.

#### 2.2.1. Viral Genotyping

Viral RNA will be extracted from the NP swab sample using the QIAamp Viral RNA Kits (Qiagen, Hilden, Germany). qPCR will be used to assess SARS-CoV-2 viral load, and if viral load is sufficient for sequencing, library preparation will then be performed using the Illumina COVIDSeq Kit (Illumina, San Diego, CA, USA), and sequencing will be carried out on an Illumina NextSeq 1000 system.

#### 2.2.2. Shotgun mHTS

Both DNA and RNA of bacteria, fungi, and viruses will be extracted from the NP swab sample using the AllPrep DNA/RNA kit (Qiagen, Hilden, Germany), and the RNA will then undergo reverse transcription via the QuantiTect Reverse Transcription Kit (Qiagen, Hilden, Germany). Both DNA and cDNA libraries will be prepared using the Illumina DNA prep kit (Illumina, San Diego, CA, USA), and sequencing will be carried out on an Illumina NextSeq 1000 system.

#### 2.2.3. Host Genomics

DNA will be extracted from whole EDTA blood via the QIAamp DNA blood kit (Qiagen, Hilden, Germany), library preparation will be performed using the Illumina Truseq DNA Exome kit (Illumina, San Diego, CA, USA), and sequencing will be carried out on an Illumina NovaSeq 6000 system.

#### 2.2.4. Whole Transcriptomic Shotgun RNAseq

RNA will be extracted from whole blood (Tempus™ Blood RNA Tube (Thermo Fisher Scientific, Waltham, MA, USA) via the Tempus™ Spin RNA Isolation kit (Thermo Fisher Scientific, Waltham, MA, USA), followed by in-column DNase treatment using the RNA Clean & Concentrator™ kit (Zymo Research, Irvine, CA, USA). Library preparation will be performed using the Illumina Stranded Total RNA Prep (Illumina, San Diego, CA, USA) and sequencing will be carried out on an Illumina NovaSeq 6000 system.

#### 2.2.5. Classical Shotgun Bottom-Up Proteomic Profiling

Plasma samples will first undergo protein depletion using the TOP 14 Abundant Protein Depletion kit (Thermo Fisher Scientific, Waltham, MA, USA), using a ratio of 500 µL depletion resin to 18 µL plasma. Once depleted, samples will then be heated to 95 °C for 5 min, cooled, and 300 µL of the sample will be added to a separate LoBind Eppendorf (Thermo Fisher Scientific, Waltham, MA, USA). DTT will then be added to a final concentration of 5 mM and incubated at 56 °C for 1 h at 1000 RPM agitation (Thermomixer C). Following this, chloroacetamide will be added to a final concentration of 50 mM and incubated in the dark at room temperature for 30 min. After incubation, 100% TCA will be added to a final concentration of 15% and the sample will be vigorously vortexed (10 s) and spun down, followed by a 30-min incubation on ice. After, the tube will be centrifuged at 4000× *g* for 5 min, supernatant discarded, and three washes performed as follows: 500 µL 100% acetone added (chilled to −20 °C), sonicated at 37 kHz (pulsed) for 2 min, centrifuged at 4000× *g* for 5 min, and supernatant discarded. After three repetitions, the tube will air dry for 10 min under a fume hood to ensure all acetone is removed, and the pellet reconstituted in 75 µL TEAB 50 mM by two repetitions of sonicating for 2 min and briefly vortexing (10 s). Finally, trypsin will be added in a 1:50 protease:protein ratio and incubated overnight at 37 °C with 750RPM agitation.

Once incubated, the sample will then be split into fractions using the Pierce high pH reversed-phase peptide fractionation kit (Thermo Fisher Scientific, Waltham, MA, USA). Fractions will then be freeze-dried and resuspended in 20 µL 3.5% ACN/0.1% TFA and finally a total of 1.2 µg peptide in 8 µL of buffer will be analyzed by reverse phase chromatography coupled to mass spectrometry on an Orbitrap Exploris 240 system coupled with an Ultimate 3000 RSnano LC system.

#### 2.2.6. Non-Targeted Metabolomic Profiling

A volume of plasma will be added to a LoBind Eppendorf followed by 3 volumes of 100% acetonitrile (MS grade). Samples will then be vortexed vigorously for 10 s and spun-down, then incubated at −20 °C overnight. Following this, samples are then centrifuged at 10,000× *g* at 4 °C for 10 min, and the upper-phase will be transferred to a new LoBind tube and mixed gently to homogenize. Then the homogenate will be divided into four equal parts, transferred to new Eppendorf tubes and dried down on a heating block at 30 °C coupled with a nitrogen flush system. Two tubes will then be resuspended in 50% ACN/0.1% formic acid for Reverse-phase based UPLC and two tubes will be resuspended in 95% ACN/0.1% formic acid and 10 mM ammonium formate for HILIC based UPLC. Samples will then be centrifuged at 10,000× *g* at 4 °C for 5 min, after which, the supernatant will be transferred to vials ready for injection.

Once samples are ready, they will then be analyzed on a Synapt-XS Q-ToF mass spectrometer (Waters) calibrated in resolution mode, coupled with an Acquity Premier UPLC system. Reverse-phase and HILIC samples will be analyzed on the UPLC-Q-ToF system coupled with an Acquity Premier HSS T3 2.1 × 100 mm, 1.8 μm column (Waters *p*/*n* 186009468) and an Acquity Premier BEH Amide 2.1 × 100 mm, 1.7 μm column (Waters *p*/*n* 186009505), respectively. For both methods, one vial will be analyzed in positive electrospray mode, and the other will be analyzed in negative ionization mode.

### 2.3. Network Construction and Multi-Omic Integration

**Figure 2:**
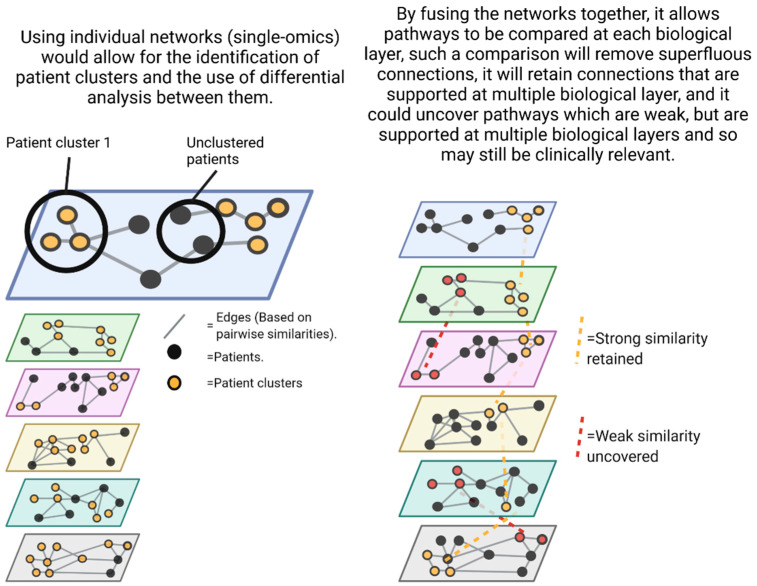
A highlight of the advantages that network analysis of multi-omic data provides, allowing us to not only remove false positives from our analysis, but simultaneously uncover false negatives that would otherwise remain unnoticed. (Created with BioRender.com©).

As data are generated, network based statistical methods (such as the nearest neighbor algorithm) will be used to construct individual networks for each -omic data described above. A visual for the proposed networks can be seen in Figure 2. Nodes will represent patients, and the edges connecting them will be based on pairwise similarities of the -omic data. This will result in patients clustering based on -omic measurements, signaling matching molecular signatures. These clusters will then be annotated with defined phenotypes and outcomes, for example, patients who did or did not develop critical illness. Differential analysis of these clusters would then identify molecules/microbes/proteins/genes presenting different patterns between groups. The final step of multi-omic integration would consist of fusing all the individual networks together. The networks will be fused by similarity network fusion (SNF) [136], and a feature ranking scheme [137] will sort the features according to their network contribution for a specified patient outcome, generating a ranking list of the most important features/pathways that can be investigated. Such an approach will not only be used to discover differences between patient groups (i.e., mild/moderate vs. severe), but will also be used to find multi-omic differences between the acute and post-COVID phases of patients, shining a new light on potential causes or biomarkers of the post COVID sequelae.

### 2.4. Clinical Translation

As a continuation of the project, as connections are revealed through the network construction, annotation, and fusion steps, results will be examined and interpreted in the light of available clinical and fundamental literature, and will enable the generation of novel scientific hypotheses. These will be validated through either an independent testing cohort using targeted assays or through in-house in vitro infection models.

## 3. Conclusions

In this article we highlight the progresses made in the area of COIVD-19 research in the context of a multi-omic overview of the disease, discussing transcriptomics, proteomics, metabolomics, metagenomics, and host and viral genomics. We identify current gaps in the disease knowledge such as the pathogenesis of post COVID-19 condition, the link between COVID-19 induced respiratory microbiome changes and transcriptomic differences, and interactions between viral transcripts and host ncRNA, to list a few. We also identify the need for more detailed multi-omic studies in the sphere of COVID-19 research.

To bridge these gaps, we have proposed a large scale, explorative, multi-omic study of a Belgium cohort featuring 225 COVID-19 patients (split evenly between critical, severe, and mild/moderate phenotypes) and 50 control patients (25 healthy controls and 25 non-COVID-19 respiratory disease patients). We plan to investigate these patients during the acute and post-COVID phase at 6 levels of biological strata: the viral genome, the respiratory microbiome, the host genome, the blood transcriptome, the blood proteome, and the blood metabolome, using gold standard HTS and mass spectrometry technologies. The data generated will then be analyzed through a network medicine approach and new hypotheses will be generated and later validated in follow up experiments.

The aim of such a project is to thoroughly explore the multi-omic state of each patient during and after SARS-CoV-2 infection, in order to identify previously unknown characteristics, biomarkers, or consequences of COVID-19 disease, with the ultimate aim of improving patient care. Additionally, we aim to allow the protocols, bioinformatics, and logistics developed during this project to be rapidly redeployed when another pandemic-type situation arises, improving novel-disease research efficiency and allowing for rapid clinical translation.

## Data Availability

Not applicable.
